# Effect of *Lactobacillus johnsonii* Strain SQ0048 on the TLRs-MyD88/NF-κB Signaling Pathway in Bovine Vaginal Epithelial Cells

**DOI:** 10.3389/fvets.2021.670949

**Published:** 2021-08-10

**Authors:** Chao Cheng, Linchong Zhang, Junxiang Mu, Qiaozhen Tian, Yanming Liu, Xiaohong Ma, Yanru Fu, Zhiguo Liu, Zhenjun Li

**Affiliations:** ^1^College of Life Science and Technology, Jining Normal University, Jining, China; ^2^Inner Mongolia Jinyu Group Co., Ltd., Hohhot, China; ^3^Shanxi Province Datong University, Datong, China; ^4^Inner Mongolia Shuangqi Pharmaceutical Co., Ltd., Hohhot, China; ^5^Hohhot Vocational College, Hohhot, China; ^6^National Institute for Communicable Disease Control and Prevention, Chinese Center for Disease Control and Prevention, Beijing, China

**Keywords:** *Lactobacillus johnsonii*, bovine vagina epithelial cells, TLRs-MyD88/NF- κB, TLR2 and TLR4, SQ0048

## Abstract

Vaginal inflammation is a common disease of the dairy cows' reproductive tract. Lactic acid bacteria can combat purulent inflammation caused by pathogenic bacteria and regulate the NF-κB signaling pathway mediated by toll-like receptors (TLRs) in the inflammatory response. We studied the effect of *Lactobacillus johnsonii* SQ0048, an isolate with antibacterial activity, on the NF-κB signaling pathway in cow vaginal epithelial cells. The expression levels of serial effectors related to the TLRs-MyD88/NF-κB signaling pathway (TLR2, TLR4, MyD88, IKK, NF-κB, IL-1β, IL-6, TNF-α, and IL-10) were measured with real-time polymerase chain reaction (RT-PCR), ELISA, and Western blot analyses. TLR2 and TLR4 were activated by SQ0048 cells, as noted by increased mRNA expression levels of TLR2 and TLR4 in SQ0048-treated bovine vaginal epithelial cells relative to control cells (*P* <0.01). SQ0048 treatment also significantly increased MyD88 and IKK expression, and activated NF-κB in vaginal epithelial cells (*P* <0.01). In addition, SQ0048 treatment also significantly increased mRNA expression levels of IL-1β, IL-6, and TNF-α, but decreased IL-10 mRNA expression levels (*P* <0.01). These data indicate that strain SQ0048 presence can improve the immune functions of cow vaginal epithelial cells by activating TLRs-MyD88/NF-κB signaling pathways. However, further *in vivo* studies are required to confirm these findings.

## Introduction

Lactic acid bacteria (LAB) are the main bacteria in the healthy female reproductive tract of humans and other mammals such as cows (1). Uterine inflammation can cause infertility in postpartum cows, and many pathogenic bacteria cause uterine inflammation (2). The production of cytokines related to inflammation is mediated by the NF-κB signaling pathway, which also plays an important role in infection by pathogenic bacteria (3). Some pathogen molecules, such as lipopolysaccharides, can be recognized by Toll-like receptors (TLRs) and they further activate NF-κB to stimulate the production of cytokines (4).

LAB control vaginal pathogenic bacteria in the female urogenital tracts by lowering pH and decreasing levels of hydrogen peroxide, and bacteriocins (5). Previous studies have demonstrated that NF-κB signaling is a key pathway involved in the anti-inflammatory role of Lactobacillus (6). The peptidoglycan of Lactobacillus can upregulate TLR2 receptors, participate in the innate immune response, and activate the TLR4 pathway (7); it also functions as a surveillance mechanism against pathogenic bacteria in the TLR4 pathway (8, 9). Extracellular polysaccharides produced by Lactobacillus can improve antiviral immunity, stimulate TLR2 and TLR4, and promote expression of IFN-α or IFN-β in cells (10). The IKK complex can inhibit NF-κB by controlling phosphorylation of the κB signaling pathway, which regulates cytokines to suppress inflammation under the stimulation of lactic acid bacteria (11). Another study confirmed that LAB could inhibit TLR-4-linked NF-κB activation (12).

Under normal conditions, LAB dominate the vaginal flora of dairy cows (1). LAB inhibit pathogenic microorganisms, reduce purulent vaginal secretions, and improve the environment of the bovine reproductive tract (13, 14). Further, LAB can change the intravaginal environment, limit the number of pathogenic bacteria reaching the uterus, and prevent or reduce the prevalence of uterine diseases in cows (13, 15). Most studies of *Lactobacillus johnsonii* have focused on the human relevance of these bacteria, and there are few reports of *Lactobacillus johnsonii* in cows. The lactobacillus strain SQ0048 isolated from bovine vagina has been shown to exhibit specific adherence to the epithelium and to produce inhibitory substances; however, the underlying mechanisms remain unclear (16). Therefore, we explored its regulatory effects and determined the probiotic mechanism of SQ0048 at the molecular level in bovine vaginal epithelial cells.

## Materials and Methods

### *Lactobacillus johnsonii* SQ0048 Source and Culture Conditions

The *Lactobacillus johnsonii* SQ0048 strain was isolated, identified, and preserved from the vaginas of healthy cows by Inner Mongolia Shuangqi Pharmaceutical Co., Ltd., as described in the patent certificate No: CN103070890B China. SQ0048 was grown anaerobically in 80 mL de Man, Rogosa, and Sharpe (MRS, Hopebio, Qingdao, China) at 37°C for 10 h. Bacteria were then collected by centrifugation at 5,000*g* for 10 min and washed three times with phosphate-buffered saline (PBS, Gibco/Thermo Fisher Scientific, Waltham, MA, USA). Finally, SQ0048 was resuspended in Dulbecco's modified Eagle medium (DMEM)/F-12 (DMEM/F12, Gibco, Grand Island, NY, USA) medium without additional reagents to the following concentrations: 10^8^ CFU/mL, 10^9^ CFU/mL, and 10^10^ CFU/mL.

### Cell Treatment

Primary bovine vaginal epithelial cells (BVECs) were isolated, purified, cultured, and identified in accordance with the methods used previously (16). Briefly, bovine vaginal tissue fragments were washed with Dulbecco's PBS (DPBS, Gibco/Thermo Fisher Scientific, Waltham, MA, USA) containing penicillin (100 U/mL) and streptomycin (100 U/mL). The fragments were further digested using 0.1% chain protease at 4°C for 16 h, then scraped into DPBS, and centrifuged at 1,500 rpm for 5 min to harvest cells. The isolated cells were resuspended in DMEM/F-12 (Dulbecco's modified Eagle medium/Ham's F-12 nutrient medium, Gibco/Thermo Fisher Scientific, Waltham, MA, USA) containing 15% fetal bovine serum (ExCell Biology, Shanghai, China), penicillin (100 U/mL), and streptomycin (100 U/mL). The cells were then incubated in 5% CO_2_ at 37°C. The origin and purity of BVECs was verified using immunocytochemical staining. BVECs were passaged to the fourth generation. When the cells adhered to more than 90% of the bottle wall area and the density of cells was approximately 3 ×10^5^ cells/mL, they were added to DPBS, washed, and added to DMEM/F-12 medium without additional reagents for 12 h. The cells subjected to different concentrations of SQ0048 (10^8^ CFU/mL, 10^9^ CFU/mL, and 10^10^ CFU/mL) were used to evaluate different parameters of the TLRs-MyD88/NF-κB signaling pathway, such as expression of TLR2, TLR4, MyD88, IKK, NF-κB, and inflammatory factors, to further determine the influence of SQ0048 on the TLRs-MyD88/NF-κB signaling pathway.

### Experimental Group Treatment

Our experiment was divided into eight groups, involving two experiment control groups (cell control group and inhibitor control group) and six testing groups (Table 1, factors section). A group of cells not subjected to any treatment was used as the cell control group, while the inhibitor control group had only inhibitors without SQ0048. The remaining six testing groups comprised SQ0048 with or without inhibitors at different concentrations (10^8^ CFU/mL, 10^9^ CFU/mL, and 10^10^ CFU/mL) (Table 1, factors section). The experiment of expression of TLR2 and TLR4 mRNA did not add inhibitors. At least three independent experiments were performed for each analysis in the same conditions.

**Table 1:** Inflammatory factor mRNA levels.

**Group**	**Factors**	**TLR4-IN-C34 (Inhibitor)**		**Sporopollenin (Inhibitor)**		**B, PD98, 059 (Inhibitor)**		**Mesalamine (Inhibitor)**		**Caffeic acid phenethyl ester (Inhibitor)**							
		**MyD88 (Major Factors)**		**MyD88 (Major Factors)**		**IKK (Major Factors)**		**NF-κB (Major Factors)**		**IL-1β** **(Major Factors)**		**IL-6 (Major Factors)**		**TNF-α** **(Major Factors)**		**IL-10 (Major Factors)**	
		**A**	**C**	**A**	**C**	**A**	**C**	**A**	**C**	**A**	**C**	**A**	**C**	**A**	**C**	**A**	**C**
1	cell	–	–	–	–	–	–	–	–	–	–	–	–	–	–	–	–
2	cell+SQ0048 (10^8^ CFU/mL)	no! change	–	no! change	–	no! change	–	↑^**^	–	↑^**^	–	no! change	–	no! change	–	↓^**^	–
3	cell+SQ0048 (10^9^ CFU/mL)	↑^**^	–	↑^**^	–	↑^**^	–	↑^**^	–	↑^**^	–	no! change	–	↑^**^	–	↓^**^	–
4	cell+SQ0048 (10^10^ CFU/m)	↑^**^	–	↑^**^	–	↑^**^	–	↑^**^	–	no! change	–	no! change	–	↑^**^	–	↓^**^	–
		B		B		B		B		B		B		B		B	
5	cell+inhibitor	–	–	–	–	–	–	–	–	–	–	–	–	–	–	–	–
6	cell+inhibitor+SQ0048 (10^8^ CFU/mL)	no! change	no! change	no! change	no! change	no! change	no! change	no! change	↓^**^	↑##	↓^**^	↑##	↑^**^	no! change	no! change	↑##	↑^**^
7	cell+inhibitor+SQ0048 (10^9^ CFU/mL)	↑##	↓^**^	↑##	↓^**^	↑##	↓^**^	↑##	↓^**^	↑##	↓^**^	no! change	↓^**^	↑##	↓^**^	↓##	↓^**^
8	cell+inhibitor+SQ0048 (10^10^ CFU/mL)	↑##	↑^**^	↑##	↓^**^	↑##	↓^**^	↑##	↓^**^	no! change	↓^**^	↓##	no! change	↑##	↓^**^	↓##	↓^*^

A, Comparison of the first group (control cells without SQ0048 or inhibitor treatment) and other groups without inhibitor treatment; B, Comparison of the sixth group (control inhibitor group without SQ0048) and other groups with inhibitor treatment; C, Comparison between groups with and without inhibitors under the same conditions of SQ0048. ↑denotes a significant increase, ↓denotes a significant decrease. * or # indicating P < 0.05 and ** or ## indicating P < 0.01. * refers to the comparison between cells in the cell control group without any treatment and other groups exposed to different concentrations of SQ0048 without inhibitor treatment; it also refers to the comparison between the group without inhibitors and the group with inhibitors at the same concentration of SQ0048; # refers to the comparison between inhibitor control groups exposed to inhibitor but not SQ0048 and other groups exposed to inhibitor. No change indicated that there was no significant difference between groups.

### Expression of TLR2 and TLR4 mRNA

Different concentrations of SQ0048 (10^8^ CFU/mL, 10^9^ CFU/mL, and 10^10^ CFU/mL) were added to cells treated and incubated for 4 h, as described previously (16). Real-time polymerase chain reaction (RT-PCR) was used to detect the expression of TLR2 and TLR4 mRNA (17). Total mRNA was extracted and reverse-transcribed to produce cDNA to conduct RT-PCR. AxyPrep Multisource Total mRNA Miniprep Kit (Axygen Scientific, Union City, CA, USA), PrimeScript II 1st Strand cDNA synthesis kit (Vazyme, Nanjing, China), and SYBR Green Master (Rox) (Roche, Mannheim, Germany) were used in this process in accordance with the manufacturers' instructions. The amplification program was as follows: 95°C for 5 min, 40 cycles of 95°C for 10 s, 58 to 60°C for 15 s, 72°C for 20 s. The primers were designed according to the bovine TLR2 and TLR4 sequences published in the GenBank database; the accession number, genome name, and sequences are shown in Supplementary Table 1. The results were analyzed using the 2^−ΔΔCt^ (ΔΔCt = ΔCt – ΔCt_control_ and ΔCt = C t_target_ – Ct_β−actin_) method and normalized to the expression of β-actin (16).

### Expression of Inflammatory Factors' mRNA

The suitable concentration of each inhibitor used in this study was added to the treated cells and incubated for suitable periods of time in the culture dishes. The suitable concentrations and times were optimized in the early stage of the experiment (Supplementary Table 2). The dishes were then washed three times with DPBS. Different concentrations of SQ0048 (10^8^ CFU/mL, 10^9^ CFU/mL, and 10^10^ CFU/mL) were added to the dishes and incubated for 4 h (16). mRNA expression levels of MyD88, IKK, NF-κB, IL-1β, IL-6, TNF-α, and IL-10 were detected under treatment with inhibitors using the RT-PCR method as described above. The primers were designed according to the bovine MyD88, IKK, NF-κB, IL-1β, IL-6, TNF-α, and IL-10 sequences published in the GenBank database (Supplementary Table 1). The names and manufacturers of each factor inhibitor used in this study are as follows: inhibitor of TLR4 is TLR4-IN-C34, co-inhibitor of TLR2 and TLR4 is Sparstolonin B, and inhibitor of NF-κB is Caffeic Acid Phenethyl Ester (Sigma-Aldrich, St. Louis, MO, USA); inhibitor of MEKK1 is B, PD98,059, and inhibitor of IKK is Mesalamine (Selleck Chemicals, Houston, Texas, USA).

### Expression of Major Factors Protein

Same as the process described above, the treated cells were incubated separately with inhibitor, and then washed. After adding different concentrations of SQ0048, the expression levels of IL-1β, IL-6, TNF-α, and IL-10 proteins were detected in supernatants using ELISA kits [Bovine IL-6 DuoSet ELISA DY8190 and Bovine TNF-alpha DuoSet ELISA DY2279 (R&D Systems, Minneapolis, MN, USA); Bovine IL-1beta ELISA Kit ab273202 and Bovine IL-10 ELISA Kit ab277386 (Abcam, Cambridge, UK)]. Western blot analysis was used to measure MyD88, IKK, and NF-κB, protein levels, because relevant ELISA kits were not available for these proteins. Protein quantification was performed as previously described (18). Total protein was extracted using M-PER (Thermo Fisher, USA) with 1% Halt Protease Inhibitor from BVECs. Protein concentrations were determined using a Pierce BCA Protein Assay Kit (Thermo Fisher Scientific, USA). The extracted proteins were stored at −80°C. A protein sample (20 μg) was resolved on 12% SDS-PAGE gel, then blotted onto polyvinylidene fluoride membranes. The membranes were blocked with 3% BSA for 2 h at room temperature. The corresponding primary antibody was incubated overnight at 4°C. The primary antibody dilutions were as follows: MyD88 1:1000, IKK 1:1000, NF-κB 1:1000, and β-actin 1:3000. Proteins were visualized using a horse radish peroxidase (HRP)-conjugated secondary antibody for 1 h at 20–22°C. The membranes were exposed to Pierce Super signal West Femto Chemiluminescent Substrate and a high-performance chemiluminescence film. Band density was quantified using ImageJ software (National Institutes of Health, Bethesda, MD, USA). The target protein band densities were normalized to β-actin. The primary antibodies used for western blot analysis were MyD88 (D80F5) Rabbit mAb (4283S) (004283S000307252017), IKK-alpha Rabbit Ab (2682S) (002682S000510132017), P-IKK-alpha/beta (S176/180) (16A6) Rabbit mAb (2697T) (002697T001910102017), P-NF-kappaB p65 (S536) (93H1) Rabbit mAb (3033T) (003033T001609082017), and NF-kappaB p65 (D14E12) XP (R) Rabbit mAb (8242T), (008242T000909152017) (Cell Signaling Technology, Danvers, MA, USA). Secondary antibodies were goat anti-rabbit IgG HRP-linked and goat anti-mouse IgG HRP-linked (Cell Signaling Technology, Danvers, MA, USA). The primary antibodies and ELISA kits specific for bovine tissues were used.

### Statistical Analyses

The experimental data were analyzed using analysis of variance (ANOVA) in SPSS and GraphPad Prism 6. Data significance was labeled with ^*^&#, ^*^or # indicating *P* < 0.05 and ^**^ or ## indicating *P* <0.01. ^*^ refers to the comparison between the cell control group with no SQ0048 and no inhibitor treatment, and other groups with different concentrations of SQ0048 and no inhibitor treatment, as well as the comparison between groups without inhibitors and the group with inhibitors with the same concentration of SQ0048. # refers to the comparison between the inhibitor control group with inhibitors but not SQ0048 and other groups with inhibitors.

## Results

### Expression of TLR2 and TLR4 mRNA

**Figure 1:**
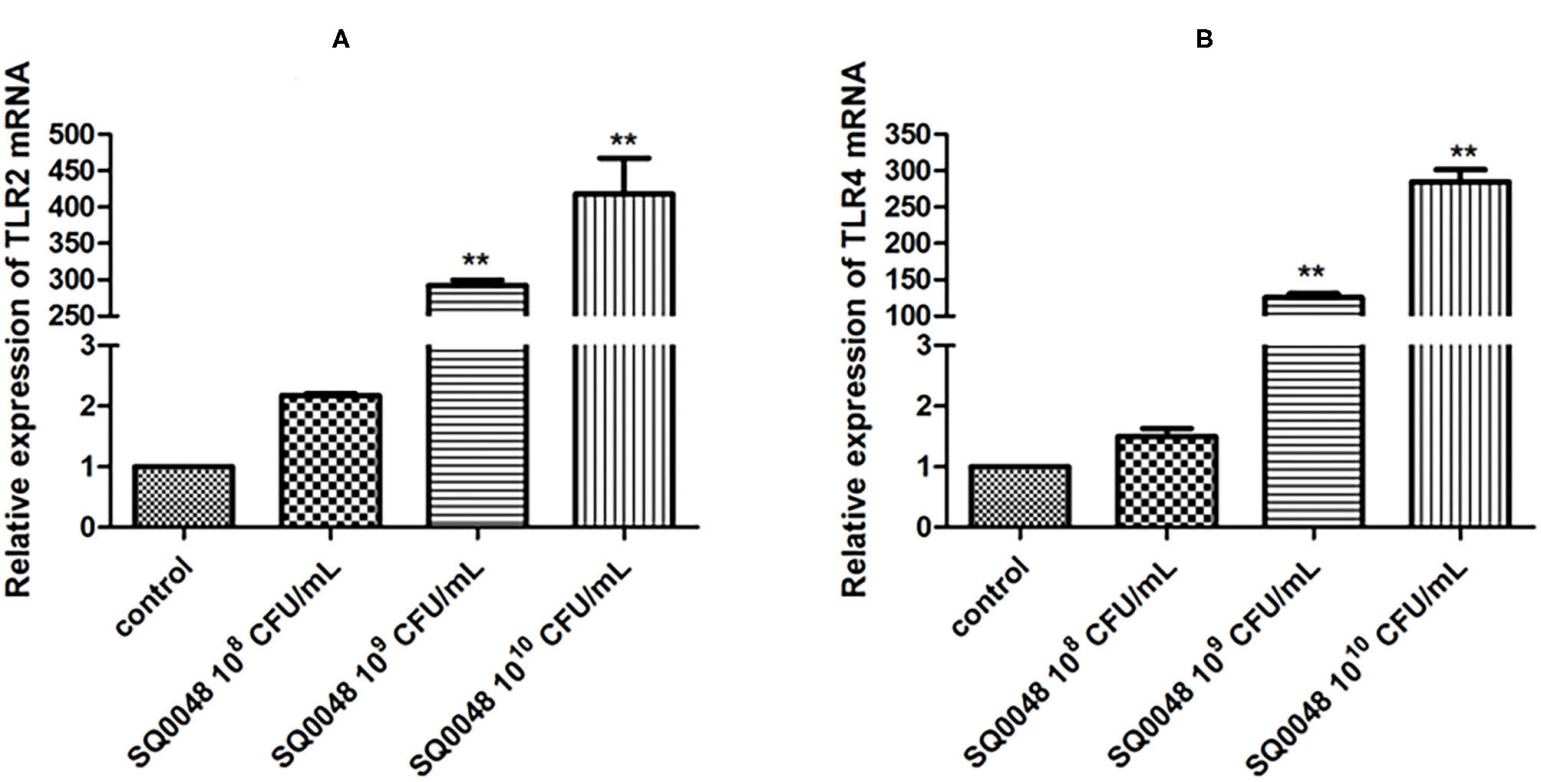
Effects of SQ0048 on mRNA expression levels of TLR2 **(A)** and TLR4 **(B)** in BVECs. All data are shown as means ± SD (*n* = 3). *P < 0.05 and **P < 0.01 relative to control.

TLR2 and TLR4 mRNA levels in the 10^9^ CFU/mL and 10^10^ CFU/mL SQ0048 groups were significantly higher than those in the cell control group (P <0.01). In contrast, the 10^8^ CFU/mL SQ0048 group did not significantly affect mRNA levels relative to cell control (Figures 1A,B).

### Inflammatory Factor mRNA Expression

**Figure 2:**
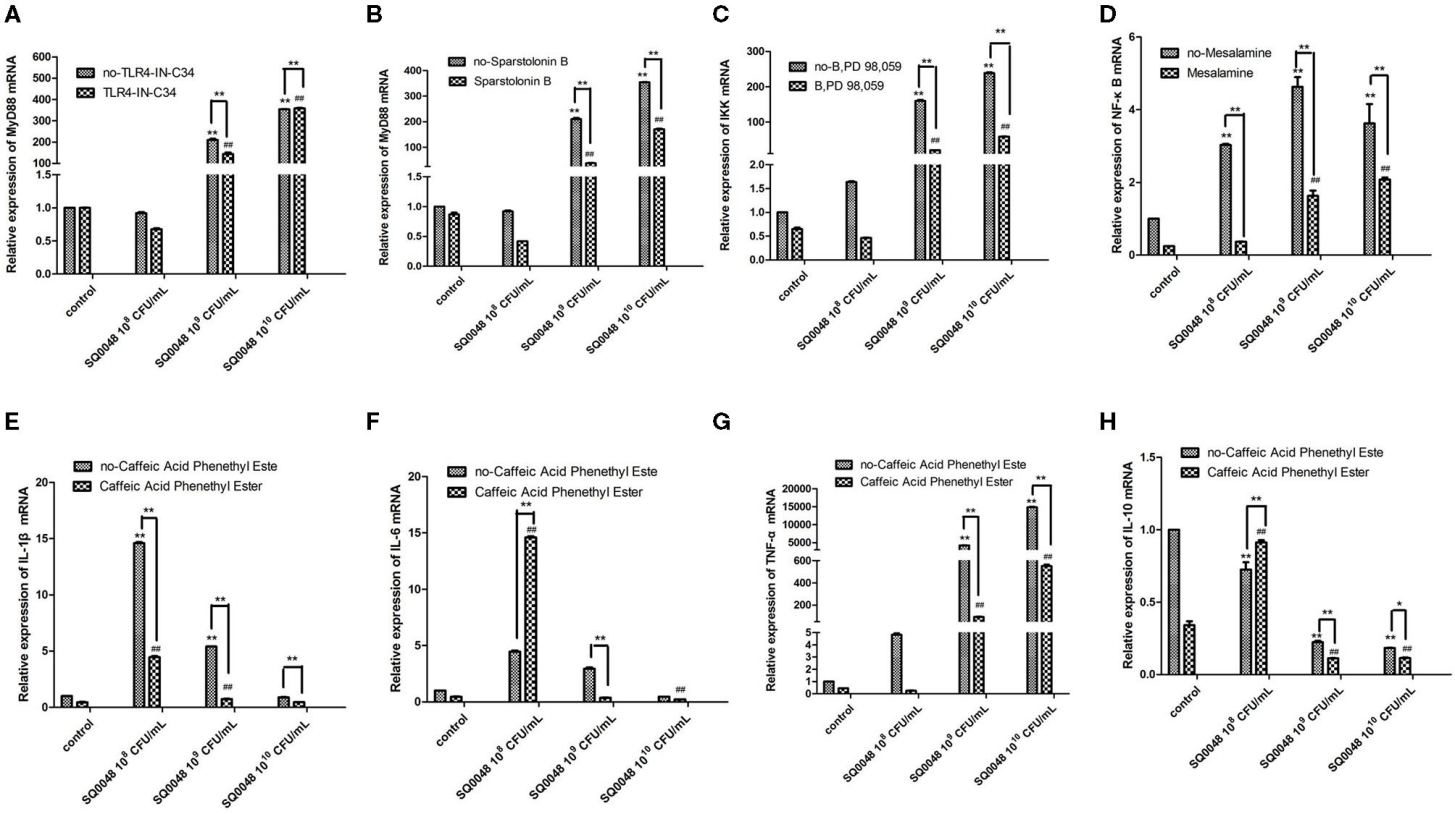
mRNA expression levels of MyD88 **(A,B)**, IKK **(C)**, NF-κB **(D)**, IL-1β **(E)**, IL-6 **(F)**, TNF-α **(G)**, and IL-10 **(H)** after treatment with inhibitors in BVECs. Note: **(A)** TLR4-IN-C34 (5 μm) for 30 min, **(B)** Sparstolonin B (10 μm) for 120 min; **(C)** B, PD98,059 (20 μm) for 90 min; **(D)** Mesalamine (1 mm) for 60 min; **(E)** Caffeic Acid Phenethyl Ester (20 μm) for 60 min; **(F)** Caffeic Acid Phenethyl Ester (20 μm) for 60 min; **(G)** Caffeic Acid Phenethyl Ester (20 μm) for 60 min; **(H)** Caffeic Acid Phenethyl Ester (20 μm) for 60 min. All data are shown as means ± SD (*n* = 3). ^*^ or # indicating *P* < 0.05 and ** or ## indicating *P* < 0.01. *refers to the comparison between cells in the cell control group not exposed to any treatment and other groups exposed to different concentrations of SQ0048 without inhibitor treatment; it also refers to the comparison between the group without inhibitors and the group with inhibitors at the same concentration of SQ0048; # refers to the comparison between the inhibitor control group with inhibitors but not SQ0048 and other groups with inhibitors.

The mRNA expression level of MyD88 in the 10^9^ CFU/mL and 10^10^ CFU/mL SQ0048 groups without inhibitors significantly increased compared with those in the cell control group (*P* <0.01) (Figures 2A,B; Table 1). The mRNA expression of MyD88 in the 10^9^ CFU/mL SQ0048 group with inhibitors (TLR4-IN-C34 or Sporopollenin) was significantly lower than that in the same concentration SQ0048 group without inhibitors (*P* <0.01). The mRNA expression of MyD88 in the 10^10^ CFU/mL SQ0048 groups with inhibitor (Sporopollenin) was significantly lower than those in the same concentration SQ0048 groups without inhibitors (Sporopollenin) (*P* <0.01) (Figures 2A,B; Table 1). The expression of IKK mRNA in the 10^9^ CFU/mL and 10^10^ CFU/mL SQ0048 groups without inhibitors was significantly higher than that of the cell control group (*P* <0.01) (Figure 2C; Table 1). The mRNA expression levels of IKK in the 10^9^ CFU/mL and 10^10^ CFU/mL SQ0048 groups with inhibitors (B, PD98, 059) were significantly lower than those in the same concentration SQ0048 groups without inhibitors (*P* <0.01) (Figure 2C; Table 1). The mRNA expression levels of NF-κB in the 10^8^ CFU/mL, 10^9^ CFU/mL, and 10^10^CFU/mL SQ0048 groups without inhibitors were significantly greater than those in the cell control group (*P* <0.01) (Figure 2D; Table 1). The mRNA expression levels of NF-κB in all three concentrations of SQ0048 with the inhibitor Mesalamine were significantly lower than those in the same concentration SQ0048 groups without Mesalamine (*P* <0.01) (Figure 2D; Table 1). IL-1β mRNA levels in the 10^8^ CFU/mL and 10^9^ CFU/mL SQ0048 groups without inhibitors were significantly increased compared with those in the cell control group (*P* <0.01), but the mRNA expression levels of IL-1βin the three concentrations of SQ0048 with inhibitors (Caffeic acid phenethyl ester) were significantly lower than those in the same concentration SQ0048 groups without inhibitors (*P* <0.01) (Figure 2E; Table 1). IL-6 mRNA levels in all concentrations of SQ0048 without inhibitors were not significantly different from those in the cell control group (*P* > 0.05). The mRNA level of IL-6 in the 10^8^ CFU/mL SQ0048 group with inhibitor (Caffeic acid phenethyl ester) was significantly higher than that in the same concentration SQ0048 group without inhibitor (*P* <0.01). The mRNA expression level of IL-6 in the 10^9^ CFU/mL SQ0048 group with inhibitor (Caffeic acid phenethyl ester) was significantly lower than that in the same concentration of SQ0048 without inhibitor (*P* <0.01) (Figure 2F; Table 1). Expression of TNF-α mRNA had the same trend as IKK (Figure 2G; Table 1), while expression IL-10 mRNA had the opposite trend (Figure 2H; Table 1).

### Inflammatory Factor Protein Levels

**Figure 3:**
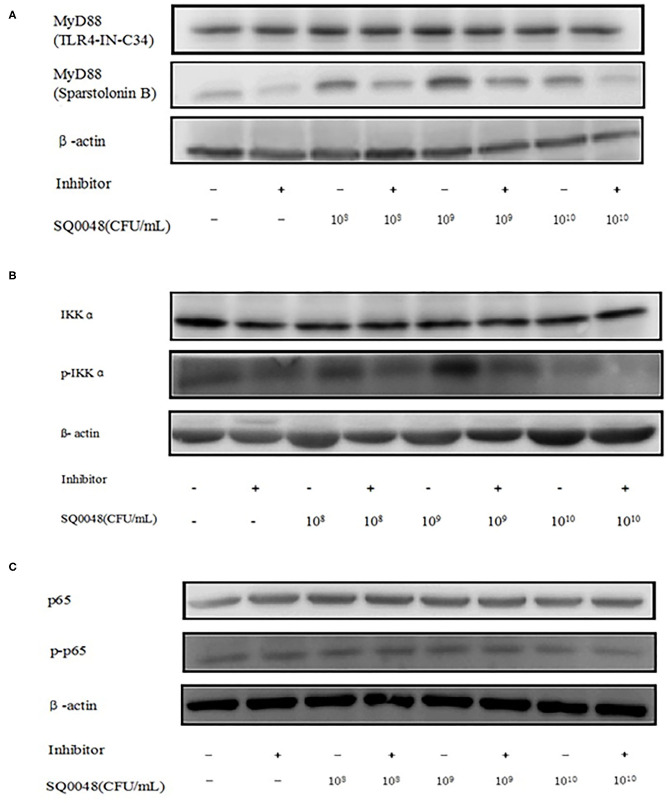
Protein expression levels of MyD88, IKK, and NF-κB after treatment with TLR4-IN-C34 and Sparstolonin **(A)**, B,PD98,059 **(B)**, and Mesalamine **(C)** in BVECs.

**Figure 4:**
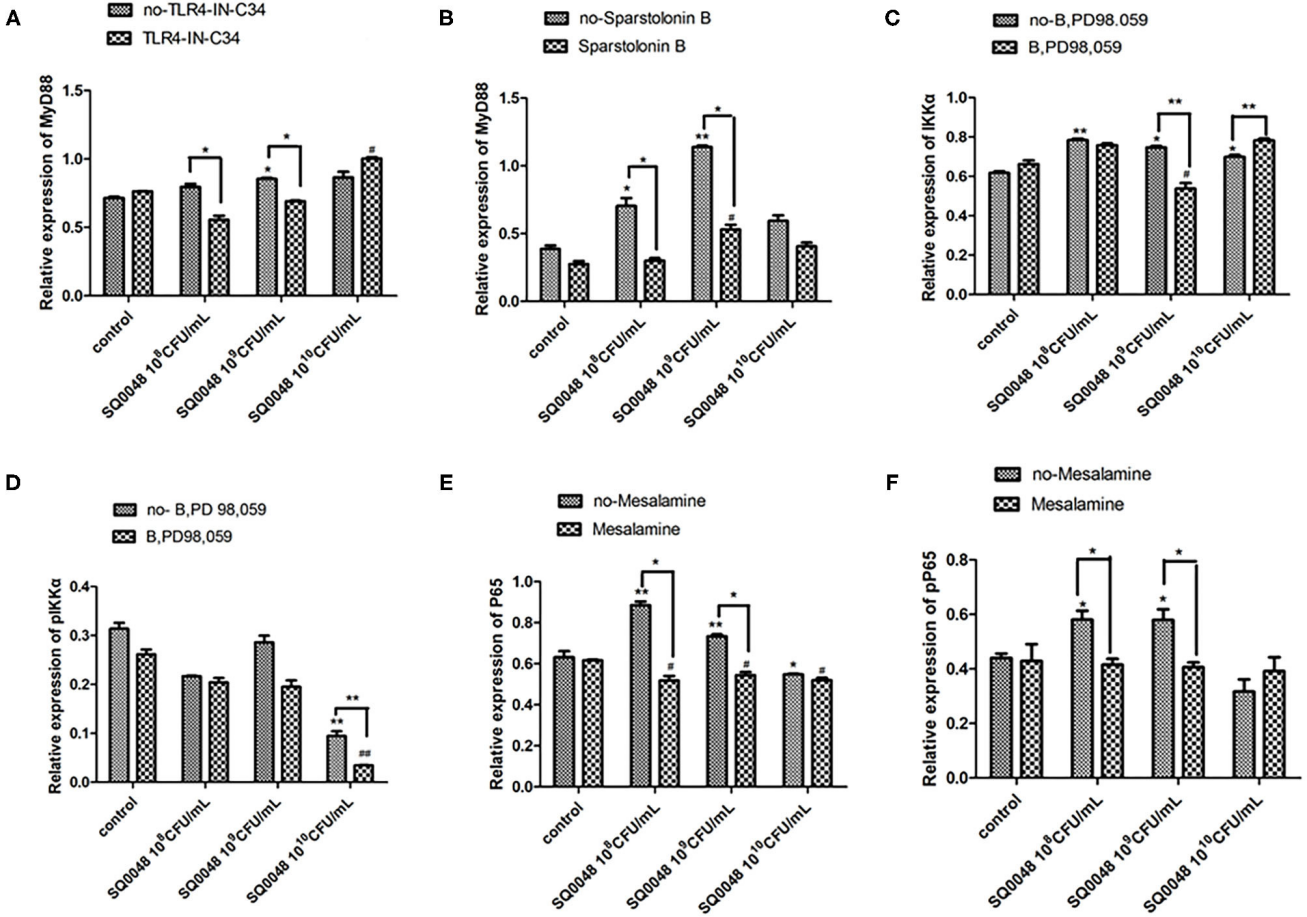
Protein expression levels of MyD88 **(A,B)**, IKK **(C,D)**, and NF-κB **(E,F)** after treatment with various inhibitors in BVECs using grayscale analysis. Note: TLR4-IN-C34 **(A)** and Sparstolonin B **(B)**; phosphorylated IKKα **(C)** and non-phosphorylated IKKα **(D)** in treatment with B,PD98,059; phosphorylated NF-κB **(E)** and non-phosphorylated NF-κB **(F)** in the treatment by Mesalamine. All data are shown as means ± SD (*n* = 3). * or # indicating *P* < 0.05 and ** or ## indicating *P* < 0.01. *refers to the comparison between cells in the cell control group not exposed to any treatment and other groups exposed to different concentrations of SQ0048 without inhibitor treatment; it also refers to the comparison between groups without inhibitors and corresponding groups with inhibitors at the same concentration of SQ0048; # refers to the comparison between cells in the inhibitor control group with inhibitors but not SQ0048 and other groups with inhibitors.

**Figure 5:**
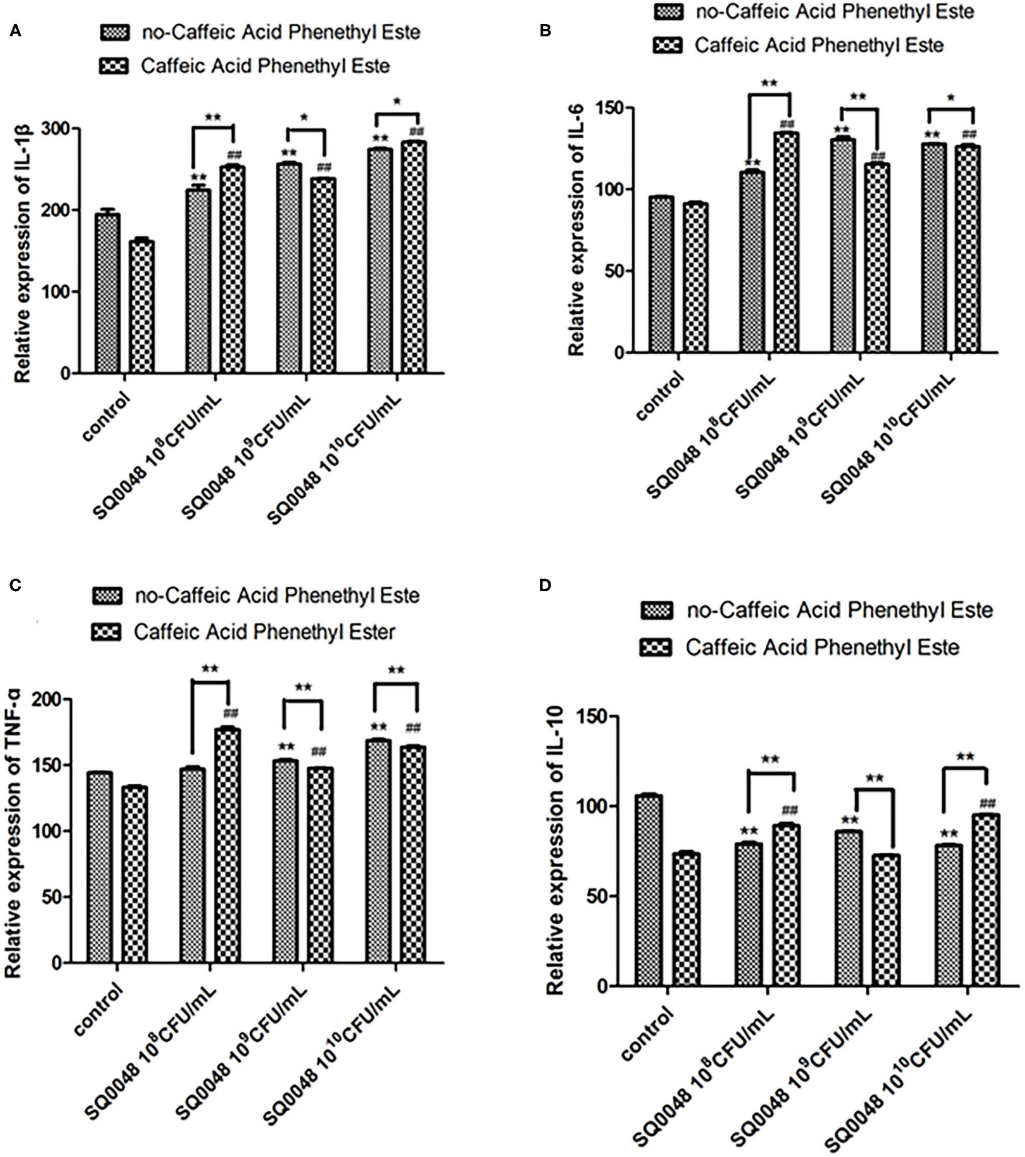
Protein expression levels of IL-1β **(A)**, IL-6 **(B)**, TNF-α **(C)**, and IL-10 **(D)** in Caffeic Acid Phenethyl Ester-treated BVECs. All data are shown as means ± SD (*n* = 3). ^*^ or # indicating *P* < 0.05 and ^**^ or ## indicating *P* <0.01. ^*^ refers to the comparison between cells in the cell control group without any treatment and other groups exposed to different concentrations of SQ0048 without inhibitor treatment; it also refers to the comparison between the group without inhibitors and the group with inhibitors at the same concentration of SQ0048; # refers to the comparison between inhibitor control groups exposed to inhibitor but not SQ0048 and other groups exposed to inhibitor.

The protein expression of MyD88 in the 10^9^ CFU/mL SQ0048 group was consistent with the mRNA expression (Figures 3A, 4A,B; Table 2). The protein expression levels of NF-κB in the 10^8^ CFU/mL and 10^9^ CFU/mL SQ0048 groups were consistent with the mRNA expression (Figures 3C, 4E,F; Table 2). The protein expression level of IKKα was similar to mRNA expression in the 10^9^ CFU/mL SQ0048 group (Figures 3B, 4C; Table 2). Phospho-IKKα levels in the 10^9^ CFU/mL SQ0048 group were not affected by the presence or absence of inhibitors (Figures 3B, 4D; Table 2). IL-1β protein secretion and mRNA expression levels were consistent in the 10^9^ CFU/mL SQ0048 groups (Figure 5A; Table 2). The protein secretion and mRNA expression of IL-6 and IL-10 were consistent in both the 10^8^ CFU/mL and in 10^9^ CFU/mL SQ0048 groups (Figures 5B,D; Table 2). TNF-α protein secretion levels and mRNA expression levels were consistent both in the 10^9^ CFU/mL and 10^10^ CFU/mL SQ0048 groups (Figure 5C; Table 2). These data indicated that 10^9^ CFU/mL SQ0048 was likely the most suitable incubation concentration for activation of the TLR-MyD88/NF-κB signaling pathway in bovine vaginal epithelial cells.

**Table 2:** Inflammatory mediator protein expression.

**Group**	**Factors**	**TLR4-IN-C34 (Inhibitor)**		**Sporopollenin (Inhibitor)**		**B, PD98, 059 (Inhibitor)**				**Mesalamine (Inhibitor)**				**Caffeic acid phenethyl ester (Inhibitor)**							
		**MyD88 (Major Factors)**		**MyD88 (Major Factors)**		**IKKα** **(Major Factors)**		**Phospho-IKKα** **(Major Factors)**		**P65 (Major Factors)**		**Phospho-P65 (Major Factors)**		**IL-1β** **(Major Factors)**		**IL-6 (Major Factors)**		**TNF-α** **(Major Factors)**		**IL-10 (Major Factors)**	
		**A**	**C**	**A**	**C**	**A**	**C**	**A**	**C**	**A**	**C**	**A**	**C**	**A**	**C**	**A**	**C**	**A**	**C**	**A**	**C**
1	Cell	–	–	–	–	–	–	–	–	–	–	–		–	–	–	–	–	–	–	–
2	cell+SQ0048 (10^8^ CFU/mL)	no! change	–	↑^*^	–	↑^**^	–	no! change	–	↑^**^	–	↑^*^	–	↑^**^	–	↑^**^	–	no! change	–	↓^**^	–
3	cell+SQ0048 (10^9^ CFU/mL)	↑^*^	–	↑^**^	–	↑^*^	–	no! change	–	↑^**^	–	↑^*^	–	↑^**^	–	↑^**^	–	↑^**^	–	↓^**^	–
4	cell+SQ0048 (10^10^ CFU/mL)	no! change	–	no! change	–	↑^*^	–	↓^**^	–	↓^*^	–	no! change	–	↑^**^	–	↑^**^	–	↑^**^	–	↓^**^	–
		B		B		B		B		B		B		B		B		B		B	
5	cell+inhibitor	–	–	–	–	–	–	–	–	–	–	–	–	–	–	–	–	–	–	–	–
6	cell+inhibitor+SQ0048 (10^8^ CFU/mL)	no! change	↓^*^	no! change	↓^*^	no! change	no! change	no! change	no! change	↓#	↓^*^	no! change	↓^*^	↑##	↑^**^	↑##	↑^**^	↑##	↑^**^	↑##	↑^**^
7	cell+inhibitor+SQ0048 (10^9^ CFU/mL)	no! change	↓^*^	↑#	↓^*^	↓#	↓^**^	no! change	no! change	↓#	↓^*^	no! change	↓^*^	↑##	↓^*^	↑##	↓^**^	↑##	↓^**^	no! change	↓^**^
8	cell+inhibitor+SQ0048 (10^10^ CFU/mL)	↑#	no! change	no! change	no! change	no! change	↑^**^	↑*##*	↓^**^	↓#	no change	no! change	no! change	↑##	↑^*^	↑##	↓^*^	↑##	↓^**^	↑##	↑^**^

A, Comparison of the first group (control cells without SQ0048 or inhibitor treatment) and other groups without inhibitor treatment; B, Comparison of the sixth group (control inhibitor group without SQ0048) and other groups with inhibitor treatment; C, Comparison between groups with and without inhibitors under the same conditions of SQ0048. ↑denotes a significant increase, ↓denotes a significant decrease. *or # indicating P < 0.05 and ** or ## indicating P < 0.01. * refers to the comparison between cells in the cell control group without any treatment and other groups exposed to different concentrations of SQ0048 without inhibitor treatment; it also refers to the comparison between the group without inhibitors and the group with inhibitors at the same concentration of SQ0048; # refers to the comparison between inhibitor control groups exposed to inhibitor but not SQ0048 and other groups exposed to inhibitor. No change indicated that there was no significant difference between groups.

## Discussion

Reproductive tract infections are often accompanied by systemic fluctuations of inflammatory cytokines (19). Vaginal epithelial cells are a part of the innate defense against vaginal inflammation. The inflammatory response of vaginal epithelial cells primarily involves the TLR and cytokine receptor pathways (20). The probiotic properties and antibacterial characteristics of LAB are closely related to the NF-κB signaling pathway (21). In the present study, 10^9^ CFU/mL and 10^10^ CFU/mL SQ0048 stimulated TLR2 and TLR4 receptors. This suggested that SQ0048 could be recognized by TLR2/4, which is consistent with previous reports (22), The specificity of TLR2/TLR6 stimulation was confirmed with blocking antibodies. The MyD88-dependent pathway primarily mediates and activates NF-κB/AP-1. Treatment with MyD88 inhibitors decreased expression of NF-κB, but not all of the LAB decreased NF-κB activation (22). LAB or their extracts and metabolites trigger the innate immune response and activate NF-κB by stimulating TNF-α signaling in epithelial cells (23). LAB strains can also increase levels of proinflammatory cytokines, such as TNF-α, IL-12, and IL-8, thereby improving host immune function (24). *Lactobacillus acidophilus* can improve the phagocytic ability of macrophages in aged mice, activate the NF-κB pathway, and regulate cytokine levels (25). Activated and inactivated *Lactobacillus rhamnosus* GG can increase macrophage function by activating NF-κB (26). Our experimental data showed that 10^9^ CFU/mL SQ0048 significantly increased expression of MyD88 and IKK and activated NF-κB in dairy cow vaginal epithelial cells. These results indicate that the biological function of SQ0048 is related to activation of the TLRs-MyD88/NF-κB signaling pathway in vaginal epithelial cells.

IL-1β is either not expressed or only weakly expressed in vaginal epithelial cells under normal physiological conditions. However, under pathological conditions, IL-1β expression increases, activating the inflammatory response. Locally high concentrations of IL-1β also contribute to killing pathogenic microorganisms and reducing their pervasion. IL-1β-mediated inflammation could have evolved to fight microorganisms and support tissue repair (27). IL-6 induces inflammation by stimulating the secretion of IL-1β. During infection, increased IL-6 levels promote migration of neutrophils to the site of inflammation and reduce leukocyte apoptosis.

Probiotic bacteria can trigger production of multiple cytokines at the systemic level, such as IL-6, IL-12, TNF-α, and IFN-γ (28). IL-6 can stimulate antibody synthesis through B cell activation and promote lymphocyte proliferation, both of which are beneficial to Th2 differentiation (29). Th2 cytokines can affect IgA, IgG, and natural killer cells, and can also identify and kill infected cells, stimulate epithelial cell proliferation, regulate intestinal and lung wound repair and healing, and inhibit mucosal fungi and bacterial infection (30).

Supernatant cultures of *Lactobacillus kefiri* BCRC14011, *Lactobacillus kefiranofaciens* BCRC16059, *L. kefiranofaciens* M1, *L. kefiri* M2, *Leuconostoc mesenteroides* M3, and *Lactococcus lactis* M4 can increase macrophage expression of IL-12, IL-6, IL-1β, and TNF-α in macrophages *in vitro*, and have strong antibacterial activity (31). *L. crispatus* and *L. iners* can increase expression of IL-6, IL-8, and TNF-α (32). TNF-α is an important cytokine involved in inflammation and immune responses. When TNF-α expression increases, it can lead to disturbances in the immune regulation of the body and contribute to pathological immune damage (33). However, an appropriate increase in TNF-α can also prevent infection. IL-10 inhibits proinflammatory responses and limits pathological inflammatory tissue damage, and is the most important anti-inflammatory cytokine (34).

Both proinflammatory cytokines and anti-inflammatory cytokines have a dual role. Proinflammatory cytokines are essential for initiation of the inflammatory response. Although an excessive inflammatory response could lead to sepsis and other related diseases, the cytokine cascade could be beneficial to the host by initiating an appropriate inflammatory response to infection or injury. This most likely occurs in humans with weakened immune systems, such as in newborns and in the elderly. Our results showed that 10^9^ CFU/mL SQ0048 significantly increased expression of IL-1β, IL-6, and TNF-α and decreased expression of IL-10. This might indicate that the anti-inflammatory mechanism of SQ0048 involves the high concentrations of IL-1β, IL-6, and TNF-α, and the low concentration of IL-10 contributes to killing pathogenic microorganisms.

We showed that 10^9^ CFU/mL SQ0048 upregulated expression levels of MyD88, IKK, and NF-κB factors by activating TLR2 and TLR4, thereby activating the TLR2/TLR4-mediated NF-κB signaling pathway. SQ0048 also increased IL-1β, IL-6, and TNF-α expression and decreased expression of IL-10. Based on our findings, we suggest that the SQ0048 strain is involved in regulating the immune function of cow vaginal epithelial cells. Activation of TLR2 and TLR4 could control the replication of pathogenic bacteria (9), and NF-κB might be related to the immunoregulatory characteristics of LAB as well as the species and source of the strain in epithelial cells (35). As described above, these data suggest that strain SQ0048 may be important for improving the immune function of cow vaginal epithelial cells, but this study has some limitations. Specifically, a mechanism for how SQ0048 improves immune function in vaginal epithelial cells is not currently apparent from these results. Consequently, in vivo experiments are needed to support the observations reported here. Nevertheless, this study provides new insights into the regulatory effects of LAB on diseases of the reproductive tracts of cows.

## Data Availability Statement

The original contributions generated for this study are included in the article/Supplementary Material, further inquiries can be directed to the corresponding author/s.

## Ethics Statement

All animal experiments were carried out according to the Animal Welfare and Research Ethics Committee of Jining Normal University (Inner Mongolia, China) (Approval ID: 41, 010, 620–1), and all efforts were made to minimize animal suffering. All animals were owned by private farms and we also obtained informed consent to use the animals in our study from the owner (s) of the animals. Written informed consent was obtained from the owners for the participation of their animals in this study.

## Author Contributions

CC performed experiments research and participated in the design of the study. ZLi and XM managed the study. CC, LZ, JM, QT, and YF conducted data processing and analyses. CC wrote the paper, ZLiu and YL critically revised the manuscript. All authors read and approved the final manuscript.

## Conflict of Interest

LZ is employed by the company Inner Mongolia Jinyu Group Co., Ltd. YL and XM are employed by the company Inner Mongolia Shuangqi Pharmaceutical Co., Ltd. The remaining authors declare that the research was conducted in the absence of any commercial or financial relationships that could be construed as a potential conflict of interest.

## Publisher's Note

All claims expressed in this article are solely those of the authors and do not necessarily represent those of their affiliated organizations, or those of the publisher, the editors and the reviewers. Any product that may be evaluated in this article, or claim that may be made by its manufacturer, is not guaranteed or endorsed by the publisher.

## Abbreviations

BV: Bacterial vaginosis
VVC: Vulvovaginal candidiasis
HPV: Human papillomavirus
HIV: Human immunodeficiency virus
LAB: Lactic acid bacteria
PCR: Polymerase chain reaction
RT-PCR: Real-time polymerase chain reaction
ECL: Enhanced chemiluminescence
TLR: Toll-like receptor.
