# Supplementary material for: Effect of Lactobacillus johnsonii Strain SQ0048 on the TLRs-MyD88/NF-κB Signaling Pathway in Bovine Vaginal Epithelial Cells
Source: Front Vet Sci. 2021 Aug 10;8:670949. doi: 10.3389/fvets.2021.670949 (PMC8383737; doi:10.3389/fvets.2021.670949)
Supplement: Supplementary file 1 [file Table_1.docx]

| Accession No. | Gene name | Sizes (bp) | Primer sequence (5’-3’) |
| --- | --- | --- | --- |
| XM_005210586.4  XM_024995446.1 | TLR4 | 101 bp | FP: TGCCTTCACTACACGGGACTTT  RP: TGGGACACCACGACAATAAC |
| XM_027513488.1 | TLR2 | 187bp | FP: CGATGACTACCGCTGTGACTC |
|  |  |  | RP: CCTTCCTGGGCTTCCTCTT |
| NM_001014382.2 | MyD88 | 94 bp | FP: ACGGTCAGACACGCACAACTT |
|  |  |  | RP: CTGCTACTGCCCCAGCGATAT |
| [NM_174353.2](https://www.ncbi.nlm.nih.gov/entrez/viewer.fcgi?db=nucleotide&id=31342236) | IKK | 83 bp | FP: AGGACGCTGTTGAGGTTGTTG  RP: GACATTGTGGTGAGCGAGGAC |
| NM_001076409 | NF-κB | 115 bp | FP: TGAGGTCCATCTCCTTCGTCT  RP: CATCCTGATCTTGCCTATTTG |
| NM_174093.1 | IL-1β | 195 bp | FP: AGGTGGTGTCGGTCATCGT  RP: GCTCTCTGTCCTGGAGTTTGC |
| NM_173923.2 | IL-6 | 269 bp | FP: ATGCTTCCAATCTGGGTTC  RP: TGAGGATAATCTTTGCGTTC |
| NM_174088.1 | IL-10 | 94 bp | FP: TGTTGACCCAGTCTCT GCTGGA  RP: GGCATCACCTCTTCCAGGTA |
| NM _173966.3 | TNF-α | 140 bp | FP: ACGGGCTTTACCTCATCTACTC  RP: GCTCTTGATGGCAGACAGG |
| NM_173979.3 | β-actin | 256 bp | FP: CCAAGGCCAACCGTGAGAAGAT  RP: CCACGTTCCGTGAGGATCTTCA |

Table. S1 Primers used in this study.
