# Supplementary material for: Effect of Lactobacillus johnsonii Strain SQ0048 on the TLRs-MyD88/NF-κB Signaling Pathway in Bovine Vaginal Epithelial Cells
Source: Front Vet Sci. 2021 Aug 10;8:670949. doi: 10.3389/fvets.2021.670949 (PMC8383737; doi:10.3389/fvets.2021.670949)
Supplement: Supplementary file 2 [file Table_2.docx]

Table S2: The best time and concentration relationship for the inhibitor to be used

| Inhibitor name | Factors inhibited by inhibitors | Suitable concentration | Suitable time |
| --- | --- | --- | --- |
| TLR4-IN-C34 | TLR4 | 5μM | 30 min |
| Sporopollenin | TLR2& TLR4 | 10μM | 120 min |
| B, PD98, 059 | MEKK1 | 20μM | 90 min |
| Mesalamine | IKK | 1nM | 60 min |
| Caffeic acid phenethyl ester | NF-κB | 20μM | 60 min |
